# Oral TGF-βR1 inhibitor Vactosertib promotes osteosarcoma regression by targeting tumor proliferation and enhancing anti-tumor immunity

**DOI:** 10.21203/rs.3.rs-2709282/v1

**Published:** 2023-04-03

**Authors:** Sung Hee Choi, Jay Myers, Suzanne Tomchuck, Melissa Bonner, Saada Eid, Daniel Kingsley, Kristen VanHeyst, Seong-Jin Kim, Byung-Gyu Kim, Alex Y. Huang

**Affiliations:** 1 Department of Pediatrics, Case Western Reserve University School of Medicine, Cleveland, OH 44106, USA;; 2 Department of Pathology, Case Western Reserve University School of Medicine, Cleveland, OH 44106, USA;; 3 Center for Pediatric Immunotherapy at Rainbow, the Angie Fowler AYA Cancer Institute, UH Rainbow Babies and Children’s Hospital, Cleveland, OH 44106, USA;; 4 MedPacto Inc., Seoul, 06668, Republic of Korea

**Keywords:** TGF-β1, Osteosarcoma, pulmonary metastasis, tumor microenvironment (TME), c-Myc

## Abstract

Osteosarcoma (OS) is an aggressive malignant bone cancer, with refractory and metastatic disease remaining a significant challenge. Transforming growth factor-β1 (TGF-β) is a potent immune suppressive cytokine in OS and the TGF-β is increased in the sera of OS patients and this increase is associated with high-grade OS and lung metastases. Therefore, blocking TGF-β1 signaling may be a novel therapy for OS treatment. Here we show that blocking TGF-β1 signaling using TGF-βR1 inhibitor, Vactosertib, significantly inhibited OS proliferation *in vitro and in vivo*. Notably, Vactosertib inhibits c-Myc expression in the OS cells. Vactosertib increased immune effectors (IFNγ^+^CD8^+^ cells and NK cells) and inhibited immune suppressors (M2-like TAM, MDSC) in the OS tumor microenvironment. Our results suggest that inhibition of TGF-β1 signaling is an effective therapeutic strategy against OS through a multi-pronged approach that targets tumor intrinsic and extrinsic factors to achieve optimal immune-effector functions and maximal clinical response.

## Introduction

Osteosarcoma (OS) is an aggressive malignant bone sarcoma originating from the primitive mesenchymal bone-forming cells in the long bones. OS is the third most common cancer among children and pediatric and adolescent & young adults (AYA) (1–5). Approximately 20% of patients have lung metastasis at initial diagnosis and an additional 40% of patients will develop lung metastasis during the later stages of disease (6, 7). Despite having aggressive chemotherapy regimens and surgical resection as treatment options, which have significantly improved the survival of patients with non-metastatic OS, such therapeutic strategies have a limited efficacy in the treatment of metastatic or recurrent OS. The survival outcome in patients with pulmonary metastatic OS (pOS) is about 20%, a statistic that has not improved significantly over the past three decades (8). Nearly all patients who develop surgically unresectable OS invariably succumb to this devastating disease. Therefore, treating metastatic and refractory OS remains an urgent challenge which demands novel therapeutic approaches.

While pOS is a well-documented major cause of OS-related deaths, the exact molecular mechanism of this phenomenon is complicated and poorly understood (9). As OS contains extremely complex and heterogeneous chromosomal and genetic alterations, recent advances in molecular precision medicine or gene replacement therapy approaches to target OS-specific mutations will likely be challenging. The OS tumor microenvironment (TME) is composed of many host-derived cell types (e.g., immune cells, endothelial cells, fibroblasts, mesenchymal stromal cells, etc.), which together favor the development of chemoresistance in the primary tumor, as well as metastatic dissemination by generating a permissive niche at both sites. Therefore, targeting key factors secreted in the OS TME could be a promising strategy for OS therapy development.

Transforming growth factor-β (TGF-β) is one of the most potent immunosuppressive molecules secreted in the OS TME. TGF-β1 contributes to the malignant TME through the recruitment and induction of immune-suppressive myeloid and regulatory T cells, each known to dynamically suppress the function of tumor-reactive cytotoxic T cells and natural killer (NK) cells (10, 11). Previous publications have reported that one of the most important contributions of myeloid cells in the TME is increased TGF-β1 production. The subsequent TGF-β1 signaling pathway in myeloid cells has been found to be required for tumor metastasis (12). It has been reported that TGF-β1 expression is increased in the sera of OS patients compared to those of healthy donors (13) and the overexpression of TGF-β1 is observed in OS tissues (14). This increase in TGF-β1 production is correlated with high-grade OS (14, 15) and the presence of lung metastases (16, 17). Also, TGF-β1 stimulates the growth of several OS cell lines (18–21). The existing literature strongly suggests TGF-β1 is a critical cytokine that promotes OS development, arguing for a strong need to critically elucidate how TGF-β1 acts on OS cells and evaluate whether blocking TGF-β1 can be a novel therapeutic approach for treating OS. Several antibody-based therapeutic approaches to inhibit TGF-β1 signaling pathways have been attempted. Although antibodies that block the receptor for TGF-β1 are effective under certain circumstances, there are limitations to using antibodies. First, it is often difficult to control toxicity, such as cardiac toxicities. Additionally, the long half-life of antibodies, prolong their global inhibition. Therefore, a small molecule-based, orally available, titratable TGF-β1 inhibitor with low toxicity and high tolerability over a long therapeutic regiment would be ideal for cancers sensitive to TGF-β1 effects, such as OS (22).

Vactosertib is a highly selective and a potent small molecule inhibitor against Type 1 TGF-β Receptor (TGF-βR1), also known as activin receptor-like kinase 5 (ALK5), which binds at the ATP binding site on TGF-βR1. This leads to specific downstream down-regulation of the SMAD pathway. Vactosertib is orally available and has 10 times the potency (IC50=11nM) of another ALK5 inhibitor, Galunisertib (IC50=110nM) (23). Although high doses of Galunisertib over long durations caused cardiac toxicities (24), whereas TEW-7197 showed limited toxicity without cardiac toxicity, and is well tolerated with minimal side effects in adults (25). Treatment with Vactosertib reduces cancer cell migration, invasion and metastasis as demonstrated in various animal cancer models, including lung metastasis and melanoma mouse models (26, 27). In this current study, we first defined the ability of Vactosertib to inhibit OS *in vitro* and *in vivo* and investigated the mechanism(s) through which it suppresses OS tumor growth and enhances immune cell activation within the TME. We show that mouse and human OS cell growth are significantly inhibited by Vactosertib *in vitro* and *in vivo* and have uncovered Myc target genes as the most significantly regulated by TGF-β1 signaling in OS cells. Additionally, we show that pOS is inhibited by Vactosertib treatment, and we found that Vactosertib suppresses accumulation of M2-like macrophages as well as suppressor myeloid cell populations while simultaneously increasing the frequencies of NK and cytotoxic T cells as the mechanism underlying enhanced tumor immunity. Interestingly, however, co-treatment of Vactosertib and immune checkpoint blockade (anti-PD-1 / anti-PD-L1 mAb) did not show synergistic inhibitory effects on OS suppression. This novel therapeutic strategy could have a high impact on the survival of patients with refractory and metastatic OS by offering feasible clinical and translational opportunities in the near future.

## Results

### Vactosertib inhibits OS cells growth *in vitro*.

**Figure 1.**
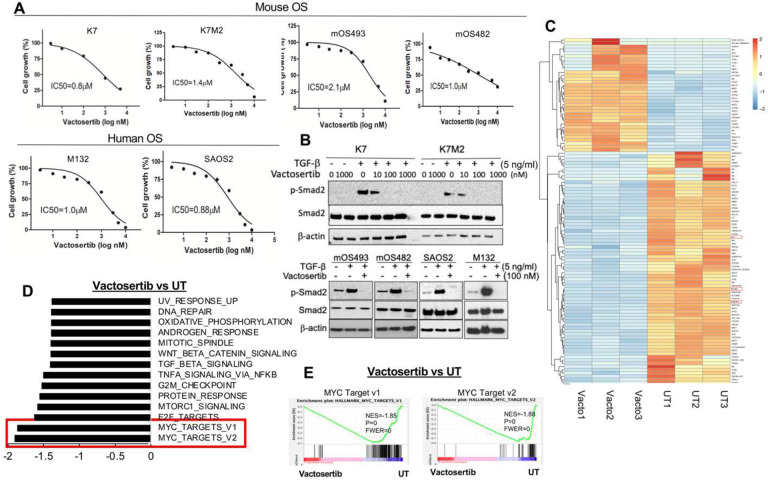
Vactosertib inhibits OS cell growth and TGF-β1 pathways. A) Effects of Vactosertib on OS proliferation. Various doses of Vactosertib (10 nM–10 μM) were incubated with mOS (K, K7M2, mOS493, and mOS482) or hOS (M132 and SAOS2). Cell growth was quantified over a 4 day period using the IncuCyte Imaging System. Nonlinear regression (curve fit) equation was calculated using GraphPad prism (N=5/group). B) Vactosertib inhibits TGF-β1 signaling pathway in OS cells. Various doses of Vactosertib (10–1000 nM) were used to treat in K7, K7M2, mOS493, mOS482, SAOS2 and M132 cells 15 minutes before TGF-β1 (5ng/ml) treatment. 1 hour after TGF-β1 treatment, cells were harvested and p-Smad2, Smad2 and β-actin expressions were measured by Western blot analysis. C-E) RNA-sequencing analysis of human OS (SAOS2) cells after Vactosertib (100 nM) or untreated (UT) for 24 hours (n=3). C) Clustered Heatmap showing the differential regulated genes identified in Vactosertib treated (Vacto) and untreated (UT) samples. These genes had an adjusted P value of <0.05 and log2 FC > 0.3 or log2 FC < −0.3. D) Hallmark Pathways from Gene Set Enrichment Analysis (GSEA) revealed dysregulated pathways in Vactosertib treated cells. P values is =0.00 and FWER is <0.05. E) GSEA enrichment plot identified downregulation of MYC target v1 and v2 pathway in Vactosertib treatment versus untreated treatment (UT).

It has been reported that TGF-β1 expressions is highly expressed in clinical OS tissue (10). Using publicly available expression cohorts, we analyzed the expression levels of TGF-β1 and its correlation with overall survival in OS patients and found that a high expression of TGF-β1 was associated with worse survival (**Suppl. Figure 1**), supporting the notion that TGF-β1 is a critical cytokine in OS disease progression and further supporting the hypothesis that effectively blocking TGF-β1 signaling can be a novel therapeutic approach for this disease. To further test this hypothesis, we investigated whether Vactosertib could directly suppress the growth of mouse (mOS) and human (hOS) OS cell lines *in vitro*. Several OS cell lines were incubated with various doses of Vactosertib (10 nM-10 μM) for 4 days, and cell growth was measured by using the IncuCyte Image System. The growth of mOS and hOS tumor cells were significantly inhibited by Vactosertib in a dose-dependent manner, with an IC50 of 0.8–2.1 μM (Figure. 1A). To verify whether the growth inhibitory effects of Vactosertib was mediated by TGF-β1 signaling inhibition, we measured TGF-β1 signaling intermediates (e.g., p-Smad2) in mOS and hOS cells. TGF-β1 induced p-Smad2 in both mOS and hOS cells was completely suppressed in the presence of 100 nM of Vactosertib (Figure. 1B). Furthermore, as little as 10 nM of Vactosertib significantly inhibited TGF-β1-induced phosphorylation of Smad2 in the mOS cells, K7 and K7M2 cells (Figure. 1B). To further understand how Vactosertib inhibits OS cell growth, we performed RNA-seq analysis of SAOS2 cells treated with Vactosertib (100 nM) for 24 hours compared to untreated cells. Vactosertib treated cells had statistically significant differential expression of 107 genes as compared to untreated samples, with 35 upregulated versus 72 downregulated genes (Figure. 1C). Among these genes, we identified significant down-regulation of Ephrin-2 (EFNB2), IL-11, and Prostate transmembrane protein androgen induced1 (PMEPA1) upon Vactosertib treatment. All three of these molecules are associated with OS tumor progression and metastasis (Figure. 1C) (28–32). Further analysis using Gene Set Enrichment Analysis (GSEA) of the RNA-seq data revealed 14 gene sets as being significantly different (p<0.001, FDR,0.05), including Wnt Beta catenin signaling, TGF-β1 and Mtorc1 signaling, all of which were significantly down regulated by Vactosertib (Figure. 1D). Notably, Myc-Targets v1 (NES=-1.85, p=0, FWER=0) and Myc-Targets v2 (NES=-1.88, p=0, FWER=0) were the most significantly inhibited by Vactosertib (Figure. 1D and E).

### Vactosertib suppresses TGF-β1-induced c-Myc signaling pathways in OS cells.

**Figure 2.**
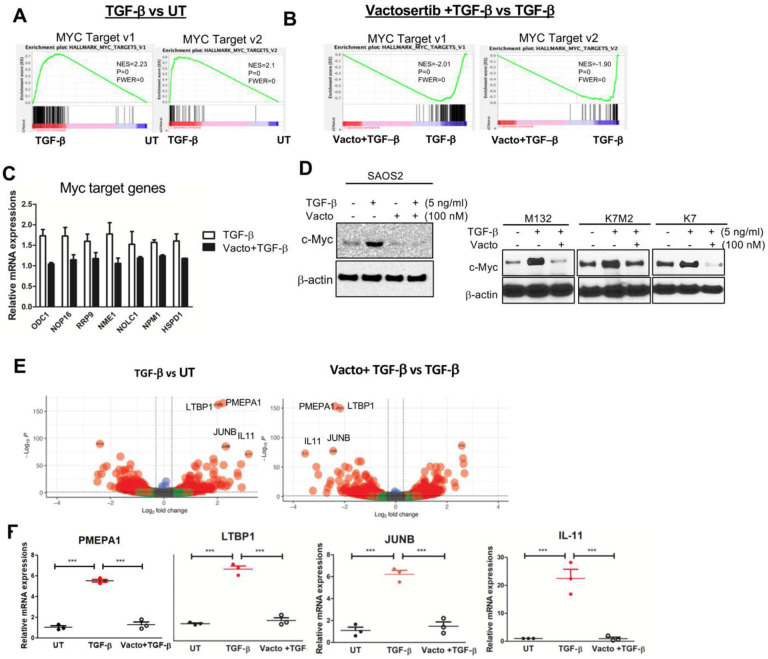
TGF-β1-induced c-Myc signaling pathways are suppressed by Vactosertib treatment in OS cells. A-B) RNA-sequencing analysis of human OS (SAOS2) cells after TGF-β1 (5 ng/ml), TGF-β1+ Vactosertib (100 nM) or untreated (UT) for 24 hours. GSEA enrichment plot of Myc target v1 and v2 pathway in TGF-β1 treatment versus untreated and in TGF-β1+ Vactosertib (Vacto) vs TGF-β1. P values is =0.00 and FWER is =0.00 for both analyses. C) mRNA expressions of Myc target genes (ODC1, NOP16, RRP9, NME1, NOLC1, NPM1 and HSPD1) in TGF-β1 or TGF-β1/ Vactosertib (Vacto) treated SAOS2 cells were measured by Realtime PCR (n=3). D) TGF-β1 (5 ng/ml) or TGF-β1 (5 ng/ml)/ Vactosertib (Vacto) (100 nM) were used to treat various OS (SAOS2, M132, K7M2, K7) for 24 hours and c-Myc and β-actin protein expressions were measured by Western blot analysis. E) Volcano plot showing the top 20 regulated genes identified by RNA-seq in TGF-β1/ Vactosertib (Vacto) vs TGF-β1. These genes have P value <0.05 and log2 FC > 0.3 and log2 FC < −0.3. F) Realtime PCR results of gene expressions of PMEPA1, LTBP1, JUNB or IL-11 in UT, TGF-β1 or Vactosertib (Vacto) +TGF-β1 treatment for 24 hours in SAOS2 cells (n=3). ***p<0.001 using a one-way ANOVA analysis followed by post-hoc Tukey’s multiple comparison tests.

Since we identified that Vactosertib inhibited Myc target genes in the RNA-seq analysis above, we investigated whether TGF-β1 could further upregulate Myc pathways in these cells, and if that up-regulation could be inhibited by Vactosertib co-treatment in OS cells. To this end, SAOS2 cells were treated with TGF-β1 (5 ng/ml) alone or co-treated with Vactosertib (100 nM) for 24 hours. GSEA analysis of the RNA-seq. dataset found the most significantly increased gene sets after TGF-β1 treatment were Myc Target v1 and v2 gene sets (p=0, FWER=0) compared with the untreated group (UT) (Figure. 2A). Conversely, Vactosertib co-treatment with TGF-β1 significantly suppressed TGF-β1 induced Myc target v1 and v2 in RNA-seq analysis (p=0, FWER=0) (Figure. 2B). Using qRT-PCR, we confirmed that the expression of Myc target genes such as ODC1, NOP16, RRP9, NME1, NOLC1, NPM1 and HSPD1 were all increased by TGF-β1 treatment and these inductions were also inhibited by Vactosertib co-treatment (Figure. 2C). TGF-β1 (5 ng/ml) treatment alone also significantly increased c-Myc protein expression in SAOS2 cells (Figure 2D). We found that low dose of Vactosertib (100 nM) completely inhibited TGF-β1induced c-Myc expression in SAOS2 cells. Moreover, Vactosertib (100 nM) treatment alone completely suppressed the total levels of c-Myc below that of baseline without exogenous TGF-β1 exposure (Figure. 2D). TGF-β1-induced c-Myc expression was confirmed in other OS cells lines, M132, K7M2 and K7 and the induction in these cell lines was also inhibited by Vactosertib (100 nM) co-treatment (Figure. 2D). Our results suggest that c-Myc regulation by TGF-β1 may be an important functional determinant of OS biology and a vulnerability that can be exploited for therapeutic purposes.

To further elucidate the role of TGF-β1 gene regulation in OS, RNA-seq. analysis was performed comparing untreated (UT), TGF-β1 single treatment, and TGF-β1 + Vactosertib co-treatment in SAOS2 cells. GO enrichment analysis of the statistically significant differentially expressed genes showed that TGF-β1 down-regulated genes involved in biological processes related to bone formation related biological processes such as ossification and extracellular matrix production **(Suppl. Figure 2A)**. Conversely, Vactosertib + TGF-β1 co-treatment yielded the opposite results **(Suppl. Figure 2C).** These results imply that TGF-β1 has a significant effect on bone formation and osteoblasts (33). We also observed that TGF-β1 up-regulated genes are associated with ribonucleoprotein complex biogenesis and ribosome biogenesis **(Suppl. Figure 2B)**, both of which were significantly inhibited by co-treatment with Vactosertib **(Suppl. Figure 2D).** Recently, c-Myc has been shown to serve as a direct up-regulator of ribosome biogenesis and therefore, c-Myc regulation by TGF-β1 may contribute to the modulation of ribosome biogenesis and cell growth (5–7). Volcano plots were used to identify genes with significantly different expression between TGF-β1 and TGF-β1 + Vactosertib co-treatment (p-value < .05 and log2 fold change > .3 or < .3.) (Figure 2E). PMEPA1, LTBP1, IL-11 and JUNB were the genes most significantly (lowest p-values) increased by TGF-β1 (Figure 2E). We additionally confirmed that the expression of these genes was suppressed by Vactosertib (Vacto) + TGF-β1 co-treatment in SAOS2 cells by qPCR analysis (Figure 2F). Previous studies have shown that these genes are involved in tumor progressions and metastasis in cancer (8–11). Furthermore, JUNB has also been reported to bind the promoter of c-Myc and regulate its expressions (12).

### Vactosertib inhibits OS growth *in vivo*.

**Figure 3.**
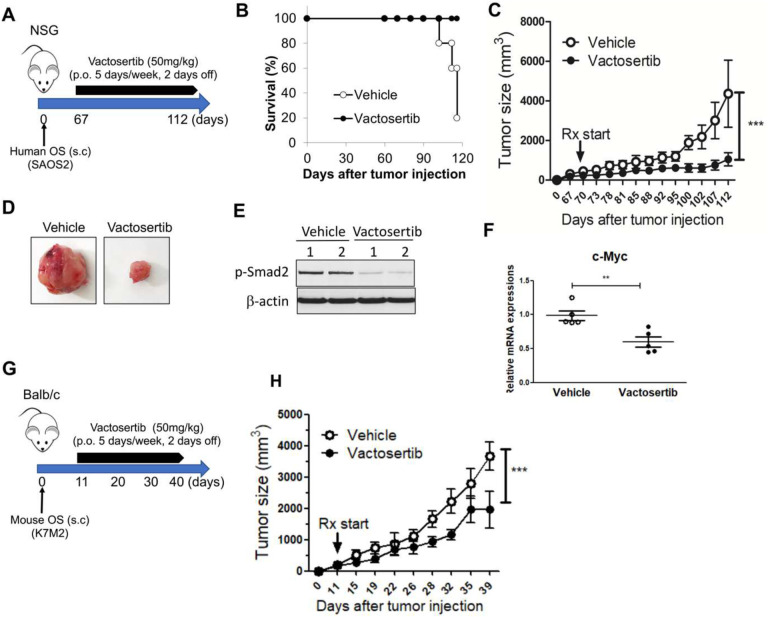
Vactosertib improves survival and inhibits human OS tumor growth *in vivo*. A) NSG mice were inoculated with 1×10^6^ human SAOS2 hOS cells (s.c.) and were treated with Vactosertib (50 mg/kg, p.o. 5 days/week), starting on day 67 after tumor injection. B) Survival rate (n=5). C) tumor sizes were measured by caliper. N=5/each group, ***p<0.001 a two-way ANOVA between groups followed by post-hoc Bonferroni’s multiple comparison tests. D) Representative tumor pictures, E) p-Smad2, β-actin expressions in tumors, F) mRNA expressions of c-Myc in vehicle or Vactosertib treated groups. N=5, **p<0.01 using an unpaired, two-tailed t-test. G) Experimental design. BALB/c mice were inoculated with 1×10^6^ K7M2 (s.c.) on Day 0, and then treated with vehicle (p.o) or Vactosertib (50 mg/kg, p.o. 5 days/week) starting on day 11. H) tumor sizes were measured by caliper. N=4, ***p<0.001 using a two-way ANOVA between groups followed by post-hoc Bonferroni’s multiple comparison tests.

To determine if Vactosertib could directly affect tumor growth in immunocompromised animals *in vivo*, NSG mice were subcutaneously injected with SAOS2 cells and treated 67 days later with Vactosertib via oral gavage (50 mg/kg, 5 days/week). Tumor growth was measured weekly and survival was also tracked (Figure 3A). By day 112, the survival rate of vehicle treated mice was only 20% however, 100% of the Vactosertib treated group survived indicating a significant increase in survival rate (Figure 3B). Additionally, the tumor volume of Vactosertib treated mice (average tumor volume 1,048 mm^3^) was significantly reduced compared with that of vehicle treated mice (average tumor volume 4,364 mm^3^) (Figure 3C and D). Furthermore, diminished lung metastasis was observed in NSG mice treated with Vactosertib **(Suppl. Figure 3A).** Western blot analysis also demonstrated that p-Smad2 (Figure 3E) as well as c-Myc mRNA expression (Figure 3F) were significantly inhibited in Vactosertib treated tumors *in vivo* (Figure 3F).

To examine the effects of Vactosertib in an immunocompetent mouse model, the mOS cell line, K7M2 was injected s.c. into BALB/c mice. On day 11 after tumor injection, mice were treated with Vactosertib (50 mg/kg, 5 days/week, p.o) and tumor volume was measured by external calipers (Figure 3G). Vactosertib treatment significantly inhibited K7M2 tumor growth (average volume 1,962 mm^3^) compared with vehicle treatment (average volume 3,669 mm^3^) (Figure 3H). 39 days after tumor injection, the mice were euthanized, and tumors were weighed. The average tumor weight of Vactosertib treated mice (1.2 g) was significantly lower compared with that of vehicle treated mice (2.3 g) **(Suppl. Figure 3B).** FACS analysis was also performed on the tumor samples to characterize the immune cell populations. No differences were observed in the percentage of the CD45, CD11b, MDSC or F480^+^ cell populations (**Suppl. Figure 3C-D).** But the percentage and total number of Arg^+^PDL1^+^ cells, which were gated on CD11b^+^F480^+^ cells, were significantly suppressed in Vactosertib treat tumors (**Suppl. Figure 3E).**

### Vactosertib inhibits pulmonary metastatic mouse OS (pOS) development *in vivo*.

**Figure 4.**
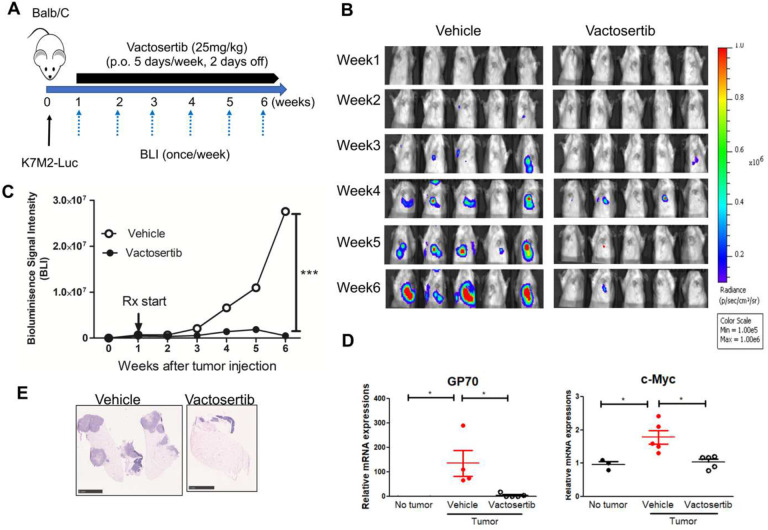
Vactosertib inhibits pulmonary metastatic mouse OS development *in vivo*. A) Experimental design. BALB/c mice were inoculated with 1×10^6^ K7M2-Luc (i.v.) on day 0, and then treated with vehicle (p.o) or Vactosertib (25 mg/kg p.o. 5 days/week) starting on day 7. B) Bioluminescence image intensity (BLI) was measured once a week. C) Graph of BLI over time. n=5, ***p<0.001 using two-way ANNOVA between groups followed by post-hoc Bonferroni’s comparison tests. D) Relative mRNA expression of GP70, and c-Myc in the lung samples of vehicle or Vactosertib treated mice on day 42 days after tumor injection compared with control lung with no tumor bearing mice. n=5/group, *p<0.05 using an unpaired two-tailed t-test. E) Representative H&E staining of lung of vehicle or Vactosertib treated lung samples day 42 day after tumor injection. Scale bar = 5 mm.

**Figure 5.**
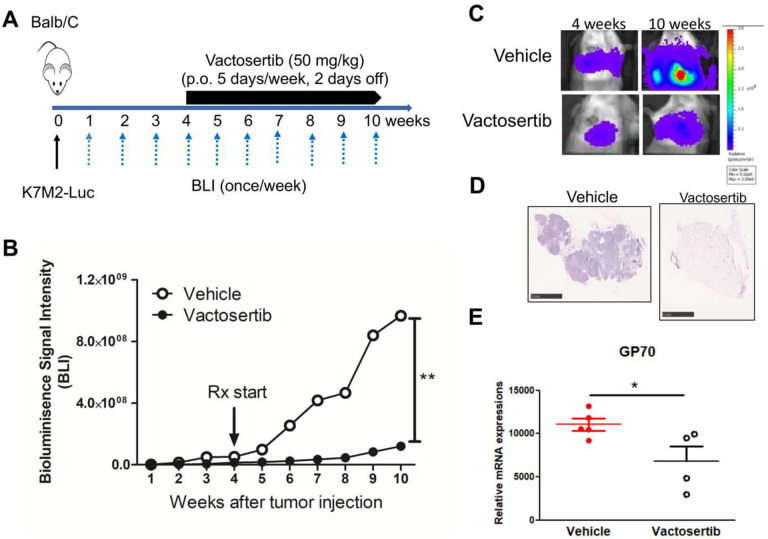
Vactosertib inhibits late stage of pulmonary metastasis *in vivo*. A) Experimental design of advanced stage of metastatic OS. BALB/c mice were inoculated with 1×10^6^ K7M2-Luc (i.v.) on day 0, and then treated with vehicle (p.o) or Vactosertib (50 mg/kg, p.o. 5 days/week) starting on day 28 (4 weeks). B) Bioluminescence image intensity was measured once a week. Graph of BLI over time. n=5, **p<0.01 using a two-way ANOVA between groups followed by post-hoc Tukey’s multiple comparison tests. C) Representative BLI images of vehicle or Vactosertib treated mice at 4 weeks and 10 weeks after tumor injection. D) Representative H & E staining of lung from mice treated with vehicle or Vactosertib 10 weeks after tumor cell injection. Scale bar = 5 mm. E) Relative mRNA expressions of GP70 in the lung samples of vehicle or Vactosertib treated tumor bearing mice compared with that of healthy mice. n=4–5/group, *p<0.05 using an unpaired two-tailed t-test.

Since, TGF-β1 production is correlated with high grade OS and associated with lung metastases (13, 14), we investigated the effects of TGF-β1 inhibition on pOS using an established pOS model in immunocompetent mice. BALB/c mice were inoculated with 1×10^6^ K7M2-Luc cells (i.v.). 7 days after tumor injection, mice were treated with Vactosertib via oral gavage (25 mg/kg once daily for 5 days with 2 days off) and monitored weekly for tumor growth by bioluminescence imaging (BLI). (Figure 4A). The average BLI of Vactosertib treated mice was 5.1×10^5^ p/sec/cm^2^/sr and that of vehicle treated mice was 2.7×10^7^ p/sec/cm^2^/sr at 6 weeks after tumor injection. Therefore, K7M2inoculated mice that were treated with Vactosertib exhibited dramatic inhibition in pOS burden (Figure 4B and 4C). This finding was further confirmed by H&E staining of lung samples (Figure 4E) and by quantitative RT-PCR (qRT-PCT) of the tumor- tumor-associated marker, Gp70 (AH1) gene transcript (34). Gp70 was significantly reduced in the Vactosertib treatment groups on day 42, an observation that correlated well with the BLI results (Figure 4B and 4D). We also confirmed that c-Myc expression in the tumor tissue was suppressed by Vactosertib treatment in these samples (Figure 4D). Together, these results showed that Vactosertib treatment significantly inhibited pOS growth *in vivo*. To further examine the antitumor effect of Vactosertib in late disease stage, we injected 1×10^6^ K7M2-luc into BALB/c mice and waited until 4 weeks post injection when pOS lesions reached an average BLI intensity of 3–5×10^7^ p/sec/cm^2^/sr. Mice were then treated with vehicle or Vactosertib (50 mg/kg p.o. for 5 days/week), and BLI was measured weekly (Figure 5A). Vactosertib was efficacious in suppressing tumor growth even when mice had a higher tumor burden (Figure 5B and 5C). This was confirmed by reduction of GP70 expression in the lungs by qRT-PCR and H&E staining in the Vactosertib treated group compared with the vehicle treated group (Figure 5D and E).

### Vactosertib increases anti-tumor immunity against pOS in TME

**Figure 6.**
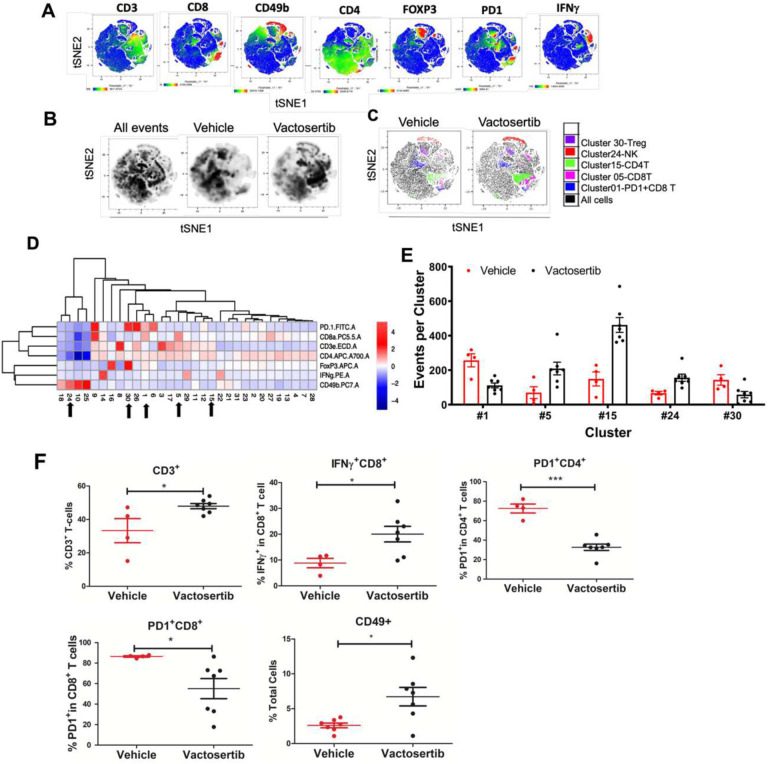
The effects of Vactosertib on tumor extrinsic T-cell profile. BALB/c mice were inoculated with 1×10^6^ K7M2-Luc (i.v).on Day 0, and then treated with vehicle (p.o) or Vactosertib (50 mg/kg, p.o. 5 days/week) starting on day 28 (4 weeks). 10 weeks after tumor injection, lung samples were collected, flow cytometry was performed and expression of CD3, CD4, CD8, CD49b, Foxp3, PD-1 and IFNγ was determined by flow cytometry. Unbiased immune cell profiling on 5000 live cells by t-Distributed Stochastic Neighbor Embedding (t-SNE) analysis was performed. A) tSNE heatmap plots show the MFI expression level and the distribution of each indicated marker. Low to high levels of protein expression are depicted in the gradient from blue (low) to red (high). B) tSNE density plots of live cells in vehicle or Vactosertib treated samples. C) tSNE dotplot overlays of clusters with significant differences (p<0.01) between vehicle and Vactosertib treatment groups. D) Heatmap showing the normalized MFI of the markers by T-cell cluster (low to high; blue to red); arrows indicate significant difference of events between vehicle and Vactosertib groups (p<0.01). E) Bar graphs showing selected clusters with significant difference of events between vehicle and Vactosertib groups (p<0.01). F) The frequency of T cell markers by conventional FACS analysis. Vehicle n=4, Vactosertib n=7 *p<0.05, ** p<0.01, using an unpaired two-tailed t-test.

To further elucidate the immune landscape within the OS TME after inhibition of TGF-β1 *in vivo*, we performed multiparametric flow cytometry with t-distributed stochastic neighbor embedding (tSNE) analysis. This analysis was performed on BALB/c mice with late-stage tumors (4 weeks after tumor injection) and treated with Vactosertib via oral gavage (50 mg/kg once daily for 5 days with 2 days off) for 6 weeks. To verify our manual gating approach of the deep profiling T cell panel and to visualize these data in two dimensions, lung samples of mice were stained with antibodies to CD3, CD4, CD8, CD49b, Foxp3, PD-1 and IFNγ and analyzed by tSNE analysis (**Suppl. Figure 4A** and Figure 6A). The generated tSNE was classified into 31 clusters (Figure 6A–D). There was a clear distinction between tSNE plots of the vehicle treated group compared to Vactosertib treated group (Figure 6B). Cluster 5 (CD8^+^CD3^+^), cluster 15 (CD4^+^CD3^+^), and cluster 24 (CD49^+^CD4^−^) were increased in the Vactosertib treated group compared with the vehicle treated group. By contrast, cluster 30 (Foxp3^+^PD1^+^CD4^+^) and cluster 1 (PD1^+^CD8^+^) populations were significantly decreased in the Vactosertib treated group compared with the vehicle treated group (Figure 6C–E). These results indicate that pharmacological inhibition of TGF-β1 can enhance CD4 T-cells, CD8 T-cells, and NK cell infiltration while suppressing Treg and PD-1^+^ cells in the OS TME. Concurrent FACS analyses also confirmed that Vactosertib treatment markedly increased IFNγ producing CD8^+^ T-cells while suppressing PD-1 expressing T cells in the K7M2 TME. These results suggest that tumor-infiltrating T lymphocytes were activated upon inhibition of TGF-β1 (Figure 6F).

**Figure 7.**
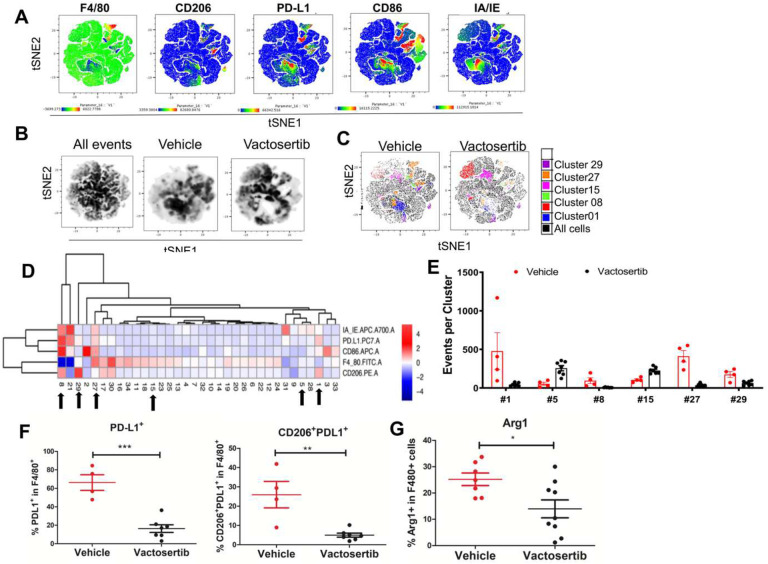
The effects of Vactosertib on tumor extrinsic myeloid cell profile. BALB/c mice were inoculated with 1×10^6^ K7M2-Luc (i.v).on day 0, and then treated with vehicle (p.o) or 50 mg/kg Vactosertib (p.o. 5 days/week) starting on day 28 (4 weeks) 10 weeks after tumor injection, lung samples were collected and examined for expression of CD45.2, CD86, CD206, F4/80, IA/IE, and PD-L1 by flow cytometry. Unbiased immune cell profiling on 5000 live CD45.2+ cells by tDistributed Stochastic Neighbor Embedding (t-SNE) analysis was performed. A) tSNE heatmap plots show the MFI expression level and the distribution of each indicated marker; low (blue) to high (red). B) tSNE density plots of CD45.2+ cells in vehicle or Vactosertib treated samples. C) tSNE dotplot overlays of clusters with significant differences (p<0.01) between vehicle and Vactosertib treatment groups. D) Heatmap showing the normalized MFI of the markers by cluster (low to high; blue to red); arrows indicate significant difference of events between vehicle and Vactosertib groups. E) Bar graphs showing selected clusters with significant difference of events between vehicle and Vactosertib groups (p<0.01). F) The frequency of myeloid cell markers by conventional FACS analysis. Vehicle n=4, Vactosertib n=7, ** p<0.01, *** p<0.001 using an unpaired two-tailed t-test. G) % of Arg1^+^F480 ^+^ cells in lung of K7M2-luc bearing mice after vehicle and Vactosertib treatment. BALB/c mice were inoculated with 1×10^6^ K7M2-Luc (i.v).on day 0, and then treated with vehicle (p.o) or 50 mg/kg Vactosertib (p.o. 5 days/week) starting on day 28 (4 weeks) 10 weeks after tumor injection, lung samples were collected and examined for expression of Arg1^+^F4/80^+^ by flow cytometry. vehicle n=7, Vactosertib n=9. *p<0.05 using an unpaired two-tailed t-test.

**Figure 8.**
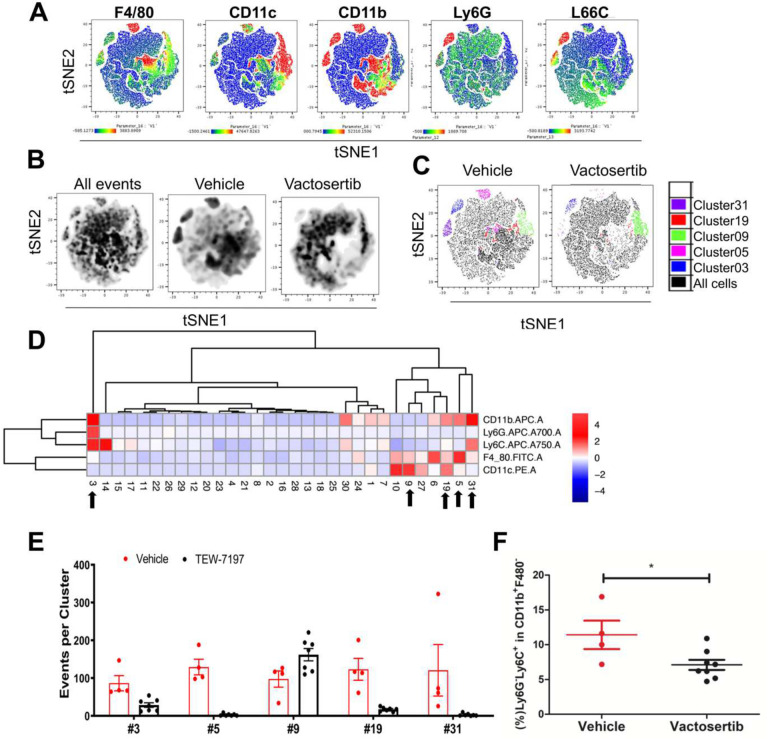
The effects of Vactosertib on tumor extrinsic MDSC cell profile. BALB/c mice were inoculated with 1×10^6^ K7M2-Luc (i.v).on Day 0, and then treated with vehicle (p.o) or Vactosertib (50 mg/kg, p.o. 5 days/week) starting on day 28 (4 weeks). 10 weeks after tumor injection, lung samples were collected and examined for expression of CD45.2, CD11b, CD11c, F4/80, Ly6C and Ly6G by flow cytometry. Unbiased immune cell profiling on 2487 live CD45.2+ cells by tDistributed Stochastic Neighbor Embedding (t-SNE) analysis was performed. A) tSNE heatmap plots show the MFI expression level and the distribution of each indicated marker; low (blue) to high (red). B) tSNE density plots of CD45.2+ cells in vehicle or Vactosertib treated samples. C) tSNE dotplot overlays of clusters with significant differences (p<0.05) between vehicle and Vactosertib treatment groups. D) Heatmap showing the normalized MFI of the markers by cluster (low to high; blue to red); arrows indicate significant difference of events between vehicle and Vactosertib groups I. groups E) Bar graphs showing selected clusters with significant difference of events between vehicle and Vactosertib groups (p<0.01). F) The frequency of MDSC cell markers by conventional FACS analysis. Vehicle n=4, Vactosertib n=7 *p<0.05, using an unpaired two-tailed t-test.

Changes in the myeloid cell populations after inhibition of TGF-β1 were also examined by tSNE analysis. Following 6 weeks of Vactosertib treatment, lung samples were separately stained with antibodies to CD45.2, CD86, CD206, F4/80, IA/IE, and PD-L1 and myeloid subsets were examined by flow cytometry (Figure 7 **and Suppl. Figure 4B**). tSNE was performed on all live CD45.2+ cells which generated 33 clusters. There were clear differences in expression profiles between the vehicle and Vactosertib treatment groups (Figure 7B and C). This approach identified a decrease in several clusters of the Vactosertib treated group compared to untreated; including clusters 1, 8, 27 and 29, which expressed CD206^+^PD-L1^+^, CD206^+^CD86^+^PDL1^+^MHCII^+^F480^−^, CD86^+^PD-L1^+^F480^+^, and CD206^+^PD-L1^+^F480^+^, respectively (Figure 7C–E). Many of these clusters express CD206 which is used as a marker for M2 macrophages. Alternately, clusters 5 and 15 which contained cells expressing MHCII^+^F480^+^ and F480^+^ were more prevalent in Vactosertib treated group than vehicle (Figure 7C–E). Conventional FACS analysis of this data confirmed the suppression of CD206^+^ PD-L1^+^ cells in lung of the Vactosertib treated mice. These results suggest that inhibition of TGF-β1 induced suppression of M2 like macrophages populations in TME of OS (Figure 7F). Furthermore, in a follow up flow cytometry experiment we found that the frequency of F4/80+ cells expressing Arg1^+^, a marker associated with tumor-associated M2-like macrophage population, was also decreased by Vactosertib treatment (Figure 7G). MDSC populations were also examined in these samples. Lung samples were separately stained with antibodies to CD45.2, CD11b, CD11c, F4/80, Ly6C and Ly6G and analyzed by flow cytometry (Figure 8A, **Suppl. Figure 4C**). tSNE analysis was performed on all live CD45.2 cells which separated into 31 clusters. 5 clusters were significantly different between vehicle treated and Vactosertib treated groups (Figure 8C–E). MDSC-like cells expressing CD11b^+^Ly6G^−^Ly6C^+^ and CD11b^+^Ly6G^+^Ly6C^+^, representing clusters 31 and 3, were decreased in the Vactosertib treated groups compared with vehicle treated group (Figure 8C–E). Furthermore, conventional FACS analysis verified that the Vactosertib treatment groups showed lower frequencies of Monocytic-MDSCs (CD11b^+^Ly6G^−^Ly6C^+^) compared with that of the vehicle treated group (Figure 8F). Taken together, tSNE analysis corroborated the finding that Vactosertib increases anti-tumor immunity against OS in TME through the accumulation of CD4^+^ T cells, CD8^+^ T cells and NK cell while suppressing the number of Tregs, MDSCs and M2-like TAMs in the OS TME.

### Co-treatment of Vactosertib and ICB (α-PD-1/ α-PD-L1 mAb) does not show synergistic effects in pOS *in vivo*.

**Figure 9.**
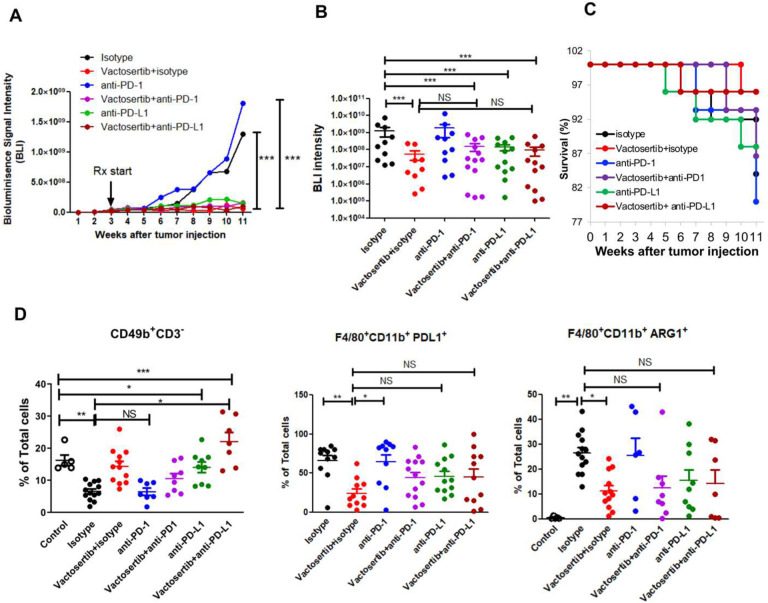
The effects of Vactosertib with ICB (α-PD-1/ α-PD-L1 mAbs) in pOS *in vivo*. A) BALB/c mice were inoculated with 1×10^6^ K7M2-Luc (i.v.) on Day 0, and then treated with Vactosertib (50 mg/kg , p.o. 5 days/week) with/without anti-PD-1 antibody or anti-PDL-1 antibody (100 ug/mice/week, i.p.) starting on day 21 (3 weeks). Bioluminescence image intensity (BLI) was measured once a week. N=9–13/group, *** p<0.001 using a two-way ANOVA B) BLI at 11 weeks after tumor injections. n=10–13, Control is the lung sample of mice without tumor injection. ***p<0.001 using an unpaired two-tailed t-test C) survival rate of isotype, Vactosertib, anti ICB antibodies after K7M2-luc injection to BALB/c mice (n=25). D) FACS analysis of NK-cell (CD49b^+^CD3^−^) and myeloid (F4/80^+^CD11b^+^PD-L1^+^, F4/80^+^CD11b^+^Arg1^+^) cells in OS TME. N=513/group, *p<0.05, ** p<0.01, *** p<0.001 using a one-way ANOVA analysis followed by post-hoc 889 Bonferroni’s multiple comparison tests.

Several studies have examined PD-L1 expression in OS cell lines and tumor tissues (34, 35) and have found a significantly high expression of PD-L1 in lung metastatic lesions which is also correlated with a poor survival rate. (36–38). Therefore, αPD-1/αPD-L1 blockade therapy in combination with Vactosertib treatment could be a potentially promising therapy for patients with advanced OS. We examined whether co-treatment with Vactosertib and αPD-1/αPD-L 1 mAb co-treatment would synergize against OS *in vivo*. K7M2-Luc cells were injected i.v. into BALB/c mice. Vactosertib (50 mg/kg p.o. 5 days/week) was administrated 3 weeks later with or without weekly ICB administration (α-PD-1 or α-PD-L1 mAb, 100ug/mouse/week, i.p.), and OS tumor burden was monitored by BLI (Figure 9A). The BLI intensity 11 weeks after tumor injection is shown in Figure 9B. Vactosertib alone inhibited OS tumor growth significantly as expected (Figure 9A and B). Although α-PD-1 antibody alone treatment did not show any therapeutic effects (Figure 9A and B). However, we did not observe any synergistic effects of Vactosertib and ICB combination treatment compared with Vactosertib alone (Figure 9B). When we compared the survival rates amongst various treatment groups, the α-PD-1 antibody treatment (80%) did not improve survival compared with isotype control treatment (84%), while Vactosertib (96%) showed protective effects on survival rate compared with isotype without demonstrating synergistic effects with either α-PD1 Ab (86%) or α-PD-L1 Ab (96%) (Figure 9C). FACS analysis was also performed on the lungs of these mice to examine immune cell populations in the metastatic TME. NK (CD49^+^CD3^−^) cells were 16.2% in control mice without tumor however, in the lungs of tumor injected mice treated with isotype control antibody, the NK population was reduced to 6.5%. When these mice were treated with Vactosertib alone or Vactosertib + α-PD-L1 co-treatment the NK population was 14.3% and 22%, respectively, which was significantly higher than that of isotype treatment (Figure 9D). PD-L1 positive and M2 like macrophage (PD-L1 F480^+^ and Arg1^+^F480^+^) which have immune suppressive activity were decreased in Vactosertib alone or combination treatment with α-PD-1 Ab (Figure 9D). But there were no statistically significant synergistic effects of Vactosertib and ICB co-treatment on induction of NK cell and suppression of M2 macrophage populations in TME compared with Vactosertib single treatment.

## Discussion

OS is an aggressive malignant bone sarcoma with a high propensity for lung metastasis. The survival outcome for pulmonary metastatic OS patients remains poor (<20% survival rate for patients with unresectable lung lesions) and accounts for most OS-related deaths. Therefore, treating metastatic OS remains a challenge and the identification of novel therapeutic strategies are desperately needed. The OS TME is infiltrated in varying degrees by both innate and adaptive immune cells including macrophages, neutrophils, dendritic cells (DCs), NK cells, T lymphocytes, and B lymphocytes. The imbalance of these cells in the OS TME is considered a key modulator of chemotherapy resistance, progression and metastasis (39). Therefore, targeting these critical factors within the OS TME would be a promising therapeutic strategy.

TGF-β1 acts as a tumor suppressor (32, 42) during the initial stages of tumor development. However, TGF-β1 signaling promotes tumor progression and metastasis in the later stages of tumor evolution (33, 43). Therefore, the role of TGF-β1 in cancer is highly complex and context dependent. In this study, we first defined that effective targeting of TGF-β1 signaling inhibition by the orally bioavailable, small molecule type 1 receptor kinase inhibitor, Vactosertib, is an effective therapeutic strategy against OS. Our multipronged approach addressed TGF-β1 inhibition in OS TME and reduced OS proliferation and metastasis via tumor-intrinsic (decreased proto-oncogene) and extrinsic immune-related factors (suppressed immune-suppressive TME) to achieve optimal conditions for enhanced immune-effector function and maximal clinical response in OS.

With regards to tumor intrinsic effects of TGF-β1 inhibition on OS cells, we found that c-Myc expression and Myc target genes were most significantly regulated by TGF-β1 signaling. c-Myc is a major proto-oncogene which promotes cell growth, cell cycle progression, transformation, apoptosis, and metabolism (40). Amplification of the c-Myc gene has been reported in OS and is a biomarker correlated with poor clinical responses and expression is seen in the most aggressive, metastatic and chemo-resistant OS phenotype (41–43). Therefore, the ability to regulate c-Myc protein expression may be an attractive approach to treat metastatic and treatment-resistant OS. Previous literatures have reported that c-Myc is down regulated by TGF-β1 stimulation in most epithelial-derived cells resulting in cell cycle arrest (44). Moreover, it is reported that downregulation of c-Myc by TGF-β is involved in the antimitogenic action of TGF-β in epithelial cells (45). Surprisingly, exposure to TGF-β1 up-regulated c-Myc expressions in both mouse OS (K7M2) and human OS (SAOS2) cells. Co-treatment with TGF-β1 and low-dose Vactosertib (100 nM) completely inhibited TGF-β1-induced c-Myc expressions and suppressed total levels of c-Myc below that of baseline without exogenous TGF-β1 exposure. Our results suggest that c-Myc regulation by TGF-β1 may be an important functional determinant of OS biology and a vulnerability that can be further exploited for future therapeutic development purposes.

Regarding the extrinsic effects of Vactosertib, we demonstrated changes in the immune cell profiles in OS TME evoked by exposure to Vactosertib. Vactosertib suppressed the accumulation of M2-like tumor-associated macrophages (TAMs) and suppressor myeloid cell populations, and simultaneously increased the frequencies and function of NK and cytotoxic T cells as the mechanism underlying enhanced tumor immunity. The innate and adaptive immune systems both play a key role in tumorigenesis and progression. In OS, it has been reported that both innate (e.g., macrophages and dendritic cells) and adaptive (e.g., T lymphocytes) immune cells participate in immune responses during OS metastasis (46–49). For instance, the imbalance of M1/ M2-polarized TAMs in favor of the M2 subtype is observed in metastatic OS (50). We identified that TGF-β1 inhibition suppressed M2-like TAM (F4/80^+^CD206^+^PD-L1^+^ and F4/80^+^Arg1^+^) populations in OS TME. Moreover, Vactosertib suppressed MDSC cells in the TME. Additionally, it has been reported that poor response of OS to chemotherapy is significantly correlated with low levels of CD8 T cells and IFNγ expression (51, 52). We observed that inhibition of TGF-β1 by Vactosertib increased IFNγ expressing CD8^+^ populations. These results support the idea that inhibition of TGF-β1 by Vactosertib has extrinsic effects on OS TME which suppresses OS tumor growth. In addition to the tumor intrinsic effects of Myc, it has recently been reported that Myc regulates the TME through its effects on innate and adaptive immune effector cells (53). Myc regulates CD47 and PD-L1 expression in multiple tumor types, including lymphoma, leukemia and liver cancer. Myc expression also correlates with PD-L1 expression (54–56). Kortlever et al. has shown that Myc activation results in the influx of macrophages alongside a loss of T cells, B cells, and NK cells and was correlated with the induction of angiogenesis (57). They also identified that Myc-induced remodeling of lung stromal cells is mediated by CCL9 (macrophage inflammatory protein-1g) and interleukin-23. Thus, MYC appears to regulate the immune response against tumors in general. In our study, we have demonstrated that inhibition of TGF-β1 suppresses c-Myc expression in OS and thus decreases the influx of M2-like TAMs and increases the infiltration of T cell and NK cells in the TME. Therefore, our results suggest c-Myc suppression through inhibition of TGF-β1 in tumor cells induces diverse stromal changes which deserves further study.

The interaction between PD-L1 and PD-1 limits antitumor function of T cells and thus possibly promotes OS metastasis by evading immune surveillance. Engagement of PD-1 and PD-L1 inhibits T-cell activation, proliferation, and cytotoxicity within the TME, resulting in an attenuated antitumor immune response. Blockade of the PD-1/PD-L1 interaction has produced favorable clinical results in numerous cancers (58, 59). In agreement with ongoing clinical observation in OS patients receiving anti-PD1 therapies (60, 61), we did not observe therapeutic efficacy with anti-PD1 mAb *in* vivo (Fig. 9). The exact reasons for this lack of clinical efficacy by targeting PD1 in OS awaits additional studies. In our studies, we observed *in vivo* efficacy of targeting PD-L1 in our mouse OS model; however, the response to anti-PD-L1 monotherapy is similar to oral Vactosertib alone (Fig. 9). Surprisingly, co-treatment of Vactosertib with ICB (anti-PD-1/anti-PDL1 mAb) did not show additive or synergistic effect compared to Vactosertib treatment alone in our OS model. Several studies have examined PD-L1 expression in OS cell lines and tumor tissues (34, 35) and have shown that there is a correlation between high expression of PD-L1 in lung metastatic lesions and poor survival rates (36–38). Vactosertib treatment of OS significantly reduced the number of PD-1^+^ T-cells and suppressed the number of PD-L1^+^ macrophages. Limiting PD-1/PD-L1 expression could be an important inhibition of TGF-β1 mediated tumor suppression mechanism, and the lack of synergistic effects of Vactosertib and ICB may be due to the loss of PD-1 or PD-L1 expression in the presence of Vactosertib. Given the superior safety and toxicity profile of oral Vactosertib in comparison to anti-PD-1 / anti-PD-L1 mAb infusions and the ease of dose titration of Vactosertib, our data supports the concept that oral Vactosertib as monotherapy is equally, if not more, effective and safe as compared to combination regimens of Vactosertib and ICB in OS.

Based on our data from this current investigation, Vactosertib has been granted an Orphan Drug Designation (ODD) in the US by the Food and Drug Administration (FDA) for the treatment of OS in August 2021. More recently, the FDA has granted an initial IND approval as well as a *Fast-Track* IND designation for the use of Vactosertib in OS. Encouraged by this, a pending multicontinent (US, Europe, Asia), multi-center phase I/II clinical trial (NCT05588648) using Vactosertib as monotherapy for the treatment of relapsed, refractory and metastatic OS is being planned for OS patients age 14 and older. This novel therapeutic agent may have a potential for high impact on the survival of pediatric and AYA patients with refractory and metastatic OS, a hard-to-treat clinical challenge plaguing the oncologists for decades. Given multiple roles TGF-β1 plays in cancer and immunobiology, the application of Vactosertib as an adjuvant in additional adaptive cellular therapy and immune modulating approaches for OS and other cancers of unmet therapeutic needs awaits thoughtful exploration.

## Methods

### Mice

Mice were housed, bred and handled under SPF conditions in the Animal Resource facilities at Case Western Reserve University. All animal experiments were performed and monitored with strict adherence to protocols in accordance with institutional guidelines and with approval of the Institutional Animal Care and Use Committee (protocol # 2016–0067) at Case Western Reserve University School of Medicine and performed in accordance with the guidelines of the American Association for Accreditation of Laboratory Animal Care and the NIH. Female BALB/c mice (6–8 weeks old, Stock#651) were obtained from Jackson Laboratory (Bar Harbor, ME). Female Nude and female NOD-Scid-Gamma (NSG) mice (6–8 weeks) are obtained directly from Case Western Reserve University Athymic Animal & Xenograft Core Facility.

### Cell lines

Mouse OS cell lines used were K7M2, K7, mOS493 and mOS482. Human OS cell lines used were M132 and SAOS2. K7 and K7M2 cells were received from Dr. Chand Khanna (NCI, NIH). mOS493 and mOS482 cells were obtained from Dr. Stuart Orkin (Boston Children’s Hospital). M132 cells are obtained from Dr. Edward Greenfield (Indiana University). SAOS2 and MG132 cells were obtained from American Tissue Type Culture Collection (ATCC, Rockville, MD). The cells were maintained in DMEM medium (Thermo Fisher Scientific, Waltham, MA) supplemented with 10% fetal bovine serum in a humidified incubator at 37C supplied with 5% CO2.

### RNA Sequencing

Constructed sequencing libraries were subject to sequencing with Illumina Novaseq^™^ 6000 platform (paired-end, 150 bp). Prior to assembly, reads containing sequencing adaptors, reads containing sequencing primers, and sequences with q quality score lower than 20 were removed. The cleaned sequencing reads were aligned to reference genome using HISAT2 package. Multiple alignments were allowed for each read sequence (up to 20 by default), with a maximum of two mismatches allowed. HISAT2 also built a database of potential splice junctions. Aligned reads of individual samples were assembled using StringTie. Transcriptomes from all samples were then merged to reconstruct a comprehensive transcriptome using a proprietary Perl script of LC Sciences (Houston, Texas, U.S.A.). Following transcriptome reconstruction, FPKM reads were evaluated by StringTie. Normalization of the raw read counts and the differential expression analyses were performed using the DESeq2 package (62). Genes were considered differentially expressed if they had an adjusted p-value (using Benjamini and Hochberg corrections) < .05 and log2 fold change > .3 or < .3. Shrunken log2 fold changes were computed using the ashr estimator from the ashr package. Genes were annotated using the org.Hs.eg.db database package (citation). The clustered heatmap was constructed using the pheatmap and RColorBrewer packages and the Volcano plots were constructed using the ggplot2 and EnhancedVolcano packages . Gene ontology (GO) enrichment was performed using the clusterProfiler package (63) with the following parameters: the Bejamini-Hochburg method to adjust the p values, an adjusted p value threshold < .05, and a qvalue < .05. The top 10 categories from each GO enrichment analysis were then plotted on dot plots. The top 10 categories from each GO enrichment analysis were then plotted on dot plots using enrichplot.

Gene Set Enrichment Analysis (GSEA) was performed using the Broad Institute tool GSEA v.2.2.1. Gene sets were tested against included the H and C7 gene sets from the Molecular Signatures Database (MSigDB).

### Mouse tumor model

OS cells were injected subcutaneously (1×10^6^ cells/0.05 ml) into the dorsal flanks of mice. Drug treatment was started when the tumors reached an average volume of 100 mm^3^. All mice were divided into two groups (n = 5 in each group), including vehicle control group (pepsin in water) and Vactosertib treatment group (50 mg/kg, p.o. 5 days/week with 2 days off). Tumor size was measured with calipers twice a week. The tumor volume (in mm^3^) was calculated using the formula: V = 1/2 (width^2^ × length). For experimental metastasis, K7M2-Luc (Luciferase+) cells (1×10^6^) were injected into BALB/c mice via the tail vein. After 7 or 28 days, mice were treated with Vactosertib via oral gavage (50 mg/kg once daily for 5 days with 2 days off) and monitored weekly for tumor growth by bioluminescence imaging (BLI). Administration of water with pepsin was used as the vehicle control. For the combination effect of Vactosertib and anti-PD-L1 or anti-PD-1 antibody, BALB/c mice were inoculated with 1×10^6^ K7M2-Luc (i.v.) on Day 0, and then treated with Vactosertib (50 mg/kg, p.o. 5 days/week) with/without anti-PD-1 antibody or anti-PDL-1 antibody (100 ug/mouse/week, i.p.) starting on day 21 (3 weeks) post tumor inoculation. BLI was measured weekly. For measuring the BLI, mice were injected i.p. with 2.25 mg of D-luciferin (GoldBio, LUCK) in PBS, anesthetized with 2.5% isoflurane, and imaged. Mice were imaged using a charge-coupled device camera–based bioluminescence imaging system (IVIS Spectrum, Caliper Life Sciences; exposure time 1–60 sec, binning 4/8/16, field of view 23 cm, f/stop 1, emission filter open). Signal was measured and recorded as total flux (photons/sec).

### Flow cytometry

Anti-mouse antibodies against several intracellular and extracellular markers were purchased along with their corresponding isotype controls from eBioscience, BD Pharmingen, ThermoFisher, or BioLegend. These include: CD3e (clone 145.2C11), CD4 (clone RM4–5), CD8α (clone 53.6.7), CD11b (clone M1/70), CD11c (clone N418), CD45.2 (clone 104), CD49b (clone MHα2), CD86 (clone GL-1), CD206 (clone C068C2), ARG1 (clone A1exF5), F4/80 (clone BM8), FoxP3 (clone FJK-16s), IA/E (clone M5/114.15.2), IFNγ (clone XMG1.2), Ly6C (clone HK1.4), Ly6G (clone 1A8), PD-1 (clone 29F.1A12), and PD-L1 (clone 10F.9G2). Additionally live/dead discrimination was done using 7-AAD (BioLegend) or Zombie NIR viability kit (BioLegend).

For staining of activated lymphocytes, isolated cell suspensions were incubated overnight *in vitro* on plate-bound anti-CD3 and anti-CD28 antibodies in 24-well plates. They were then stimulated with Cell Activation Cocktail containing Brefeldin A (BioLegend) for 5 hours before staining. All cells were washed in ice-cold FACS buffer (PBS/2.5 mM EDTA/0.5% FBS), followed by incubation with Fc blocking antibody (anti-mouse CD16/32 in FACS buffer) for 15 min at 4°C. Cells were washed and antibodies were added for 30 min on ice. The samples were washed again with ice-cold FACS buffer and then run on the BDAccuri C6 or CytoFlex flow cytometer (Beckman Coulter). The data were analyzed using BDAccuri, CytExpert or FlowJo software. Intracellular staining IFN-γ, Foxp3, and Arg1 were performed using an intracellular staining kit (eBiosciences) according to the manufacturer’s instructions.

In addition to conventional FACS analysis, high-dimensional clustering using t-distributed stochastic neighbor embedding (tSNE) was performed on the same number of live CD45.2+ events from each sample, as indicated, which was exported and clustered based on the mean fluorescent intensity (MFI) of each marker of interest by the Rphenograph package.

### Hematoxylin and eosin staining

For hematoxylin and eosin staining (H&E), lung tissues were washed with PBS and fixed in 10% formalin. Samples were embedded in paraffin wax, sectioned, stained with H&E, and examined by light microscopy.

### Western blotting

For Western blot, tumor or lung samples were lysed by incubation in lysis buffer (150 mM NaCl, 20 mM Tris-Cl, pH 7.5, 1 mM PMSF, 1 mM Na3VO4, 25 mM NaF, 1% aprotinin, 10 μg/ml leupeptin) on ice for 30 min. 20 μg aliquots of proteins were separated by electrophoresis in 10% SDS/PAGE mini gels and transferred to nitrocellulose membrane (Invitrogen). Following blocking, membranes were incubated in buffer containing the primary antibody, followed by washing and incubation for 1hr at room temperature with horseradish peroxidase-conjugated secondary antibodies. Immunostaining was visualized by ECL.

### Quantitative RT-PCR analysis.

Total RNA was isolated from tissues using Trizol reagent (Invitrogen). For reverse transcriptionPCR (RT-PCR), cDNA was synthesized using a High Capacity cDNA synthesis kit (Applied Biosystems). Quantitative RT-PCR was performed using BioRad CFX96 Real-Time System C1000 Thermal Cycler. ΔΔC_T_ was calculated using β-actin as control gene with samples normalized to expression in wild type tissue. Quantification was performed using instrument software.

### Statistical analysis

Two-tailed, unpaired Student’s *t*-test was used to compare differences between groups. Two-way ANOVA was applied to evaluate isotype, Vactosertib, anti-PD-1, Vactosertib and anti-PD-1, antiPD-L1 and Vactosertib+anti-PD-L1 groups. Bonferroni’s *post hoc* test was performed when applicable. All data are presented as the means ± standard error of the mean (SEM). Statistical significance was accepted to be a *p*-value less than or equal to 0.05, with *p<0.05, **p<0.01, ***p<0.001. All statistical analyses were performed using GraphPad Prism (GraphPad Software, Inc., La Jolla, CA, USA).

## Data Availability

The authors confirm that the data supporting the findings of this study are available within the article and its supplementary materials. The reagents are available from the corresponding author, A.Y.H., upon reasonable request and full execution of a Material Transfer Agreement agreed to by both parties.
